# Whole genome microarray analysis in non-small cell lung cancer

**DOI:** 10.1080/13102818.2014.989179

**Published:** 2015-01-19

**Authors:** Mohammad AL Zeyadi, Ivanka Dimova, Vladislav Ranchich, Blaga Rukova, Desislava Nesheva, Zora Hamude, Sevdalin Georgiev, Danail Petrov, Draga Toncheva

**Affiliations:** ^a^ Department of Medical Genetics, Faculty of Medicine, Medical University of Sofia, Sofia, Bulgaria; ^b^ Clinic of Thoracic Surgery, Medical University of Sofia, Sofia, Bulgaria

**Keywords:** non-small cell lung cancer, array CGH, oncogenes, tumour-suppressor genes

## Abstract

Lung cancer is a serious health problem, since it is one of the leading causes for death worldwide. Molecular–cytogenetic studies could provide reliable data about genetic alterations which could be related to disease pathogenesis and be used for better prognosis and treatment strategies. We performed whole genome oligonucleotide microarray-based comparative genomic hybridization in 10 samples of non-small cell lung cancer. Trisomies were discovered for chromosomes 1, 13, 18 and 20. Chromosome arms 5p, 7p, 11q, 20q and Хq were affected by genetic gains, and 1p, 5q, 10q and 15q, by genetic losses. Microstructural (<5 Mbp) genomic aberrations were revealed: gains in regions 7p (containing the epidermal growth factor receptor gene) and 12p (containing *KRAS*) and losses in 3p26 and 4q34. Based on high amplitude of alterations and small overlapping regions, new potential oncogenes may be suggested: *NBPF4* (1p13.3); *ETV1*, *AGR3* and *TSPAN13* (7p21.3-7p21.1); *SOX5* and *FGFR1OP2* (12p12.1-12p11.22); *GPC6* (13q32.1). Significant genetic losses were assumed to contain potential tumour-suppressor genes: *DPYD* (1p21.3); *CLDN22*, *CLDN24*, *ING2*, *CASP3*, *SORBS2* (4q34.2-q35.1); *DEFB* (8p23.1). Our results complement the picture of genomic characterization of non-small cell lung cancer.

## Introduction

Lung cancer is a leading cause of cancer-related death all over the world. It is classified into two types: small-cell lung cancer (SCLC) and non-small cell lung cancer (NSCLC).[1] NSCLC is more common and makes up about 85% of all lung cancers.[2] Although the majority of NSCLC is caused or induced by cigarette smoking, 10% of the patients are non-smokers.[3] Lung carcinogenesis – like the development of other cancers – is a multistage process and involves alterations in multiple genes and diverse pathways. Inherited polymorphisms in a number of genes, especially in carcinogen-metabolising genes, affect the individual susceptibility to development of lung cancer.[4,5] More frequent changes include chromosomal rearrangements, microsatellite instability, deregulated expression of telomerase and alterations in angiogenesis. Mutational activation of oncogenes and inactivation of tumour-suppressor genes, and subsequent increased genetic instability are major genetic events in lung carcinogenesis (reviewed in [6–8]). By the time lung cancer is clinically diagnosed, as many as 10–20 genetic alterations may have accumulated.[9]

High-resolution microarray-based comparative genomic hybridization (array CGH) is a modern molecular technology used for performing a complete scan of genomic DNA. It demonstrates the presence of genetic losses and/or increased copy numbers of the genetic material in the tumour tissue and is characterized by high sensitivity and reliability of the results. The method is widely used for screening genomic analysis in a large number of malignancies.

In this study, we performed whole genome microarray analysis for screening of copy number changes in NSCLC in order to assess the genomic instability in different stages of this tumour type.

## Subjects and methods

### Subjects

In the microarray study, we used DNA isolated from tumours of 10 patients diagnosed with primary NSCLC. The clinical characteristics of the patients are summarized in Table 1. Six patients were male and four, female. The average age of the patients was 61 years (ranging from 52 to 67 years).

**Table 1.** Clinical data about the analysed lung cancer cases.

Tumour No.	Gender	Age	TNM stage	Stage	Histotip	Metastases in the lymph nodes
Т1	Woman	66	pT1N0M0, G2	IB	AC	**−**
Т2	Woman	67	pT2N2M0, G3	IIIA	AC	**+**
Т3	Man	52	pT2N1M0, G2	IIB	AC	**+**
Т4	Man	58	pT2N0M0, G3	IB	AC	**−**
Т5	Woman	54	pT2N0M0, G3	IB	SCC	**−**
Т6	Man	65	pT2N0M0, G2-3	IB	SCC	**−**
Т7	Man	59	pT4N2M0, G2	IV	SCC	**+**
Т8	Man	67	pT2N1M0, G2	IIIA	SCC	**+**
Т9	Man	53	pT2N0M0, G2-1	IB	SCC	**−**
Т10	Woman	67	pT1N0M0, G2	IB	AC	**−**

### Materials

The materials were taken after tumour resection in the Department of Thoracic Surgery ‘St. Sofia’ during the period November 2007–December 2009 and were kept in the tissue bank of the Department of Medical Genetics. The collection of samples was approved by the Institutional Ethics Committee of the Medical University of Sofia and all participants signed informed consent forms. All tumours were staged postoperatively according to the classification system of the International Union Against Cancer.[10] Among the 10 tumours with NSCLC, five had the histology of adenocarcinoma (AC) and five, of squamous cell carcinoma (SCC).

### DNA extraction

Total DNA was extracted from the tissue samples, using QIAamp DNA minikit (Qiagen, Hilden, Germany). DNA concentration was measured spectrophotometrically by using a NanoDrop® ND-2000 spectrophotometer working with volumes of 1–2 μL. The 260/280 ratio was in the range of 1.8–2.0 for each sample. As an additional quality control, DNA was checked in a 1% agarose gel: DNA of high molecular weight (>50 kbp) indicated it suitable for use.

### Comparative genomic hybridization on DNA microarrays

The principle of microarray-based comparative genomic hybridization is based on competitive hybridization between alternatively labelled tumour and normal DNA on slides with spotted DNA sequences (oligonucleotides or bacterial artificial chromosomes (BAC)). We used genomic array CytoChip (BlueGnome, Cambridge, UK) Oligonucleotide microchips 2×105 K with a density of 105.072 sequences covering the entire genome with a resolution of 35 Kb. CytoChip Oligo microchips have two separate fields for hybridization, allowing for simultaneous analysis of two samples on one chip. Each chip has a unique code at the bottom of the glass.

### Array CGH probe labelling, hybridization, image capture and data analysis

The control and reference DNA was labelled by random priming, using Blue Gnome Fluorescent Labelling System. The labelled products were purified by AutoSeqTM G50 columns. The labelling mix was added in each tube containing DNA and primers; test DNA was labelled by fluorochrome Cy3 and control DNA, by Cy5. Hybridization was done by dissolving precipitated probes in hybridization buffer. Arrays were washed in standard saline citrate solutions with decreasing concentrations and scanned by a GenPix 4100A. All data were processed with the program BlueFuse Multi version 2.2 (BlueGnome, Cambridge). In data processing, base 2 logarithm (log2) ratios of Cy3 and Cy5 intensities are generated for all hybridized clones. Normal copy numbers are considered to be in the range of −0.3 to +0.3; values above +0.3 were evaluated as gain/amplification and those under −0.3, as losses (deletions). Genomic profiles were represented with logarithmic ratios on the *Y*-axis and along the 23 chromosomes on the *X*-axis. Individual chromosomal profiles were represented with clone positions on the *Y*-axis and logarithmic ratios on the *X*-axis.

### Statistical analysis

Contingency table analysis and χ^2^ test were used to assess the relationship between gene copy number changes and tumour phenotype, i.e. tumour stage. *P* < 0.05 was considered as statistically significant.

## Results and discussion

### Array CGH and copy number aberrations in lung cancer

Lung cancer is a serious health problem because it is one of the leading causes of cancer mortality worldwide. To the best of our knowledge, we report for the first time the results from whole genome array CGH analysis in Bulgarian patients diagnosed with primary NSCLC. Array CGH is the most powerful tool for genetic screening of tumours.[11,12] Its ability to simultaneously detect DNA copy number changes at multiple loci over the whole genome and to provide high-resolution mapping of variation in copy numbers was used in our study. Candidate genes responsible for disease can be identified; thus, the results could lead to new discoveries or could confirm the current data.[13] They could help in the better understanding of the mechanisms of the disease by revealing potential oncogenes and tumour-suppressor genes located in aberrant regions revealed in our patients.

Our array CGH results showed that the average number of pathological aberrations per tumour was 10.1, among which genetic losses were prevalent. The average copy number loss per tumour was 5.8 and the average copy number gain per tumour was 4.3. The most frequent aberrations detected in our study were genetic gains of 7p (containing the epidermal growth factor receptor gene *EGFR*) and 12p (containing *KRAS*) and genetic losses of 3p26 and 4q34. Genetic losses were more frequent than gains in our study. In other studies, there are different data: some report prevalence of genetic losses,[12,14] while others, of genetic gains.[15,16]

### Analysis of large and regional (greater than 5 Mbp) aberrations

**Figure 1.**
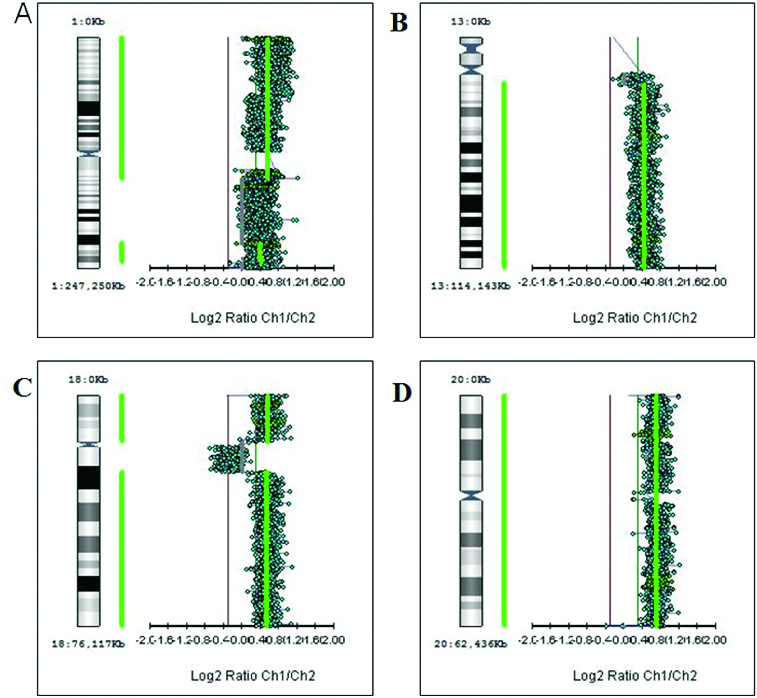
Large aberrations such as gains of whole chromosomes (trisomies): trisomy 1 (A), trisomy 13 (B), trisomy 18 (C) and trisomy 20 (D).

**Figure 2.**
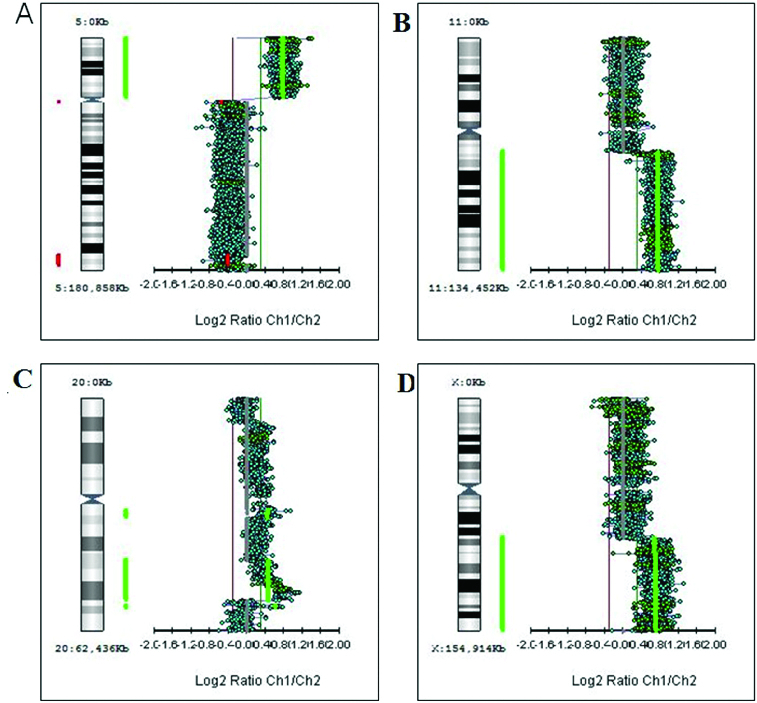
Genetic aberrations involving chromosome arms. (A) Gain of the short arm of chromosome 5 (5p+). (B) Gain of the long arm of chromosome 11 (11q+). (C) Gain of the long arm of chromosome 20 (20q+). (D) Gain of the long arm of X chromosome (Xq+).

**Figure 3.**
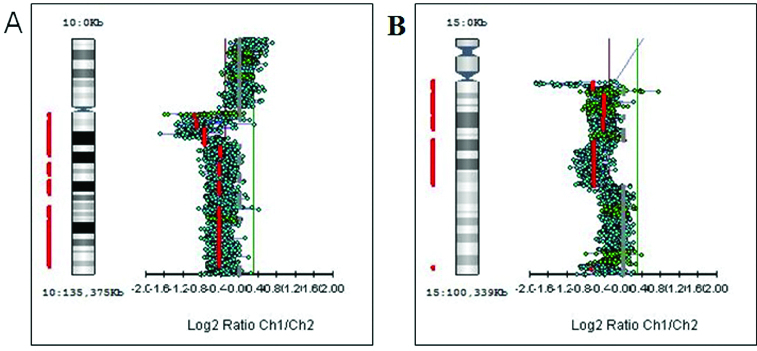
Genetic losses of chromosome arms. (A) Deletion of the long arm of chromosome 10 (10q−). (B) Deletion of the long arm of chromosome 15 (15q−).

First, we studied large aberrations involving whole chromosomes or chromosome arms. We found in different tumours additional copies of whole chromosomes (trisomies) for chromosomes 1, 13, 18 and 20 (+1, +13, +18 and +20) as shown in Figure 1. There were genetic gains for the following chromosome arms: the short arm of chromosome 5 (5p +), the short arm of chromosome 7 (7p+), the long arms of chromosomes 11 (11q+), X (Xq+), 14 (14q+) and 20 (20q+) in different tumours (Figure 2). There were genetic losses for the following chromosome arms: the short arm of chromosome 1 (1p−), the long arms of chromosomes 5 (5q−), 10 (10q−) and 15 (15q−) in different tumours (Figure 3). All these large aberrations were found in the group of early carcinomas.

The second step in our study was to analyse the so-called regional aberrations, which are larger than 5 Mbp. We observed regional genetic gains in chromosomes 4, 6, 7, 10, 11, 12, 13, 16 and X (Table 2). The highest frequency was revealed for regional gains of 7p21.3-p21.1 and 12p12.1-p11.22 – in 20% of the tumours. Regional genetic losses were found in chromosomes 1, 2, 3, 4, 5, 6, 7, 9, 11, 12, 13, 14, 15, 16, 17, 18, 19 and X (Table 3). The highest frequency was detected for regional losses of 3p26.2-p26.1 and 4q34.2-q35.1 – in 20% of the tumours.

**Table 2.** Regional genetic gains.

Tumour No.	Cytoband	Start	End	Size (bp)
3	4q11-4q21.1	52,383,888	77,575,772	25,191,884
6	6p23-6p22.3	15,459,250	20,707,670	5,248,420
9	6p21.2-6p21.1	40,027,843	45,619,656	5,591,813
10	**7p21.3 -7p21.1**	7,637,458	19,495,021	11,857,563
4	**7p21.3 -7p21.1**	11,809,793	18,729,930	6,920,137
3	10q21.1-10q22.1	55,828,450	71,348,794	15,520,345
10	11q24.1 -11q24.3	122,211,906	129,420,515.5	7,208,609.5
4	**12p12.3-12p11.22**	16,876,006	27,760,499	10,884,493
5	**12p12.1-12q13.12**	22,369,353	47,551,298	25,181,945
5	13q12.3-13q14.11	28,057,665	40,830,712	12,773,047
4	16q21-16q23.2	64,946,502	78,281,702	13,335,200
6	Xp22.33- Xp22.11	702	24,669,519	24,668,817

Note: Regions (genes) of interest are shown in bold.

**Table 3.** Regional genetic losses.

Tumour No.	Cytoband	Start	End	Size (bp)
10	1p21.3-1p13.1	98,099,261	115,748,977.5	17,649,716.5
9	2q33.3-2q37.2	204,815,208	236,711,526	31,896,318
10	**3p26.3-3p26.1**	132,210	5,599,399.5	5,467,189.5
9	**3p26.2-3p25.3**	3,219,568.5	8,962,547	5,742,978.5
9	3p14.1-3p12.3	68,559,381.5	77,661,856.5	9,102,475
9	4p16.2-4p16.1	5,109,436.5	10,722,944.5	5,613,508
3	4q25-4q26	109,765,527	115,095,866	5,330,339
3	**4q34.1-4q35.1**	172,789,678	187,179,203	14,389,525
9	**4q34.2-4q35.2**	177,514,856	190,594,214.5	13,079,358.5
3	5q35.1-5q35.3	168,992,861	176,570,197	7,577,337
5	6p12.1-6q21	56,742,923	105,360,483	48,617,560
10	7q31.31-7q32.2	119,784,951	129,658,377.5	9,873,426.5
4	9p23-9p21.1	11,894,279	30,444,221	18,549,942
10	11p15.5-11p15.2	583,612	14,914,265.5	14,330,653.5
10	12p13.31-12p13.1	8,081,123	13,574,193.5	5,493,070.5
10	12q14.1-12q14.3	60,290,737.5	65,601,505.5	5,310,768
10	12q23.3-12q24.21	104,805,666.5	114,780,044	9,974,377.5
9	13q12.11-13q12.3	19,829,534.5	28,923,152.5	9,093,618
6	14q11.1-14q21.2	18,149,503	43,040,553	24,891,050
3	15q11.1-15q13.2	18,315,236	28,865,096	10,549,860
5	16q13-16q23.2	56,488,812	80,026,359	23,537,547
9	17p13.1-17p12	9,067,635	14,324,518.5	5,256,883.5
10	18q12.2-18q12.3	34,385,712	40,144,137	5,758,425
6	19q12-19q13.2	35,531,871	46,359,194	10,827,323
5	Xp22.33-Xp21.2	1,047,758	29,798,607	28,750,849
5	Xq13.1-Xq21.1	70,198,765	83,830,128	13,631,363
10	Xq22.3-Xq24	106,985,608	116,530,057.5	9,544,449.5

Note: Regions (genes) of interest are shown in bold.

In the group of early stage cancers, the most common types of aberrations were large ones (average of two large aberrations per tumour) and regional aberrations (average 5.6 regional aberrations per tumour) as compared with advanced cancers, where we observed zero large aberrations per tumour and zero regional aberrations per tumour (*p* < 0.03). Losses of whole chromosomes or chromosome arms are found in early stages of carcinogenesis and contribute to overall genomic instability of tumours. One of the first and earliest signs of lung epithelium in NSCLC is exactly the loss of the short arm of chromosome 3,[17] which was also found in two of the tumours in our study, both from stage IB.

Another common genetic aberration characteristic of early lung carcinogenesis is deletion of chromosome 9.[17] In our study, we found loss of the short arm of chromosome 9 in a tumour from stage IB. We also observed large aberrations involving whole chromosomes (+1, +13, +18 and +20) or chromosome arms (1p−, 5p+, 5q−, 7p+, 10q−, 11q+, 14q+, 15q−, 20q+ and Xq+). There was also regional genetic loss in 1p21.3-p13.1 and high amplitude loss in the same region.

Deletions of the short arm of chromosome 1 are common among various cancers. Nomoto et al. [18] identified common unbalanced changes in 1p36 in breast cancer. This is the chromosomal region where the tumour-suppressor gene *TP73*, which shows significant homology with the *TP53* gene, is located. Liu et al. [3] examined the *TP73* gene in six NSCLC cell lines and found abnormal methylation in exon 1 and loss of expression at mRNA and protein level. The change in methylation of TP73 may play an important role in the mechanism of silencing gene expression as well.[3] In our experiments, among patients with early stage NSCLC, early genetic changes affecting 5p were identified. Here, we detected gain of 5p15.33. This locus harbours genes *TERT*, *SLC6A19* and *SLC6A18*.

### Analysis of microstructural aberrations (less than 5 Mb)

We selected the aberrations that have a high amplitude (log2 ratio T/N > 0.5 for genetic gains and <−0.5 for genetic losses). Following this approach, 42 aberrations were selected. Of these, 18 were genetic gains (Table 4) and 24, genetic losses (Table 5). There was amplification of the same locus, 1q31.3, in two of the analysed tumours (Table 4). Also in two other tumours, there was loss of a single region, 8p23.1 (Table 5).

**Table 4.** Genetic gains with size <5 Mbp and high amplitude (log2 ratio T/N > 0.5) .

Tumour No.	Cytoband	Start	End	Genes
2	1p13.3	108,727,866	108,778,747	*SLC25A24*, ***NBPF4***
7	1q25.1	172,819,907	172,950,762	–
2	1q31.3	195,011,374	195,065,896	–
6	1q31.3	195,011,374	195,065,896	–
4	2q37.3	242,514,623	242,656,003	**THAP4*,* ATG4B*,* DTYMK*,* ING5**
7	3q28	190,368,588	191,292,238	*GMNC*, *OSTN*, *UTS2D*, ***CCDC50***, *PYDC2*
6	8p12	37,400,925	38,252,355	***ZNF703***, ***ERLIN2***, *PROSC*, *GPR124*, ***RAB11FIP1***, ***BRF2***, *ADRB3*, *GOT1L1*, *EIF4EBP1*, *STAR*, ***ASH2L***, ***LSM1***, ***BAG4***, ***DDHD2*****, *PPAPDC1B*, *WHSC1L1***, *LETM2*
6	8p11.23-8p11.1	38,647,653	43,599,753	*PLEKHA2*, *HTRA4*, ***TM2D2***, ***ADAM9*****, *ADAM32*, *ADAM18*, *ADAM2*,***IDO1*, *IDO2*, ***C8orf4***, ***ZMAT4***, *SFRP1*, *GOLGA7*, ***GINS4***, *AGPAT6*, *ANK1*, *KAT6A*, *AP3M2*, *PLAT*, ***IKBKB***, *VDAC3*, *SLC20A2*, ***POLB***, ***DKK4***, *CHRNB3*, *CHRNA6*, *THAP1*, *RNF170*, *HOOK3*, *FNTA*, *SGK196*, *HGSNAT*, *POTEA*, *MIR486*, *MIR4469*
4	8q24.3	141,439,283	141,579,769	***TRAPPC9***, *CHRAC1*, *EIF2C2*
5	9p23	11,649,326	11,858,554	–
4	9q34.3	138,727,320	139,162,389	*CAMSAP1*, *UBAC1*, *NACC2*, *LHX3*, *QSOX2*
9	11p13	31,682,617.5	35,734,215	*ELP4*, *PAX6*, *RCN1*, ***WT1***, ***EIF3M***, *CCDC73*, *PRRG4*, *QSER1*, *DEPDC7*, *TCP11L1*, *CSTF3*, *HIPK3*, ***CD59***, *FBXO3*, ***LMO2***, *CAPRIN1*, *NAT10*, *ABTB2*, *CAT*, *ELF5*, *EHF*, *APIP*, *PDHX*, ***CD44***, *SLC1A2*, *PAMR1*, *MIR1343*
7	13q22.1	72,490,874	73,265,427	–
4	13q32.1	94,503,340	95,093,497	***GPC6***
7	16p13.2	6,957,553	7,062,587	*RBFOX1*
6	16p12.2	21,448,123	21,647,357	*METTL9*
5	17p13.3	1,197,564	2,535,589	***YWHAE***, ***CRK***, *INPP5K*, *MYO1C*, *PITPNA*, *SLC43A2*, *SCARF1*, *RILP*, *PRPF8*, *SERPINF2*, *TLCD2*, *WDR81*, *SERPINF1*, *SMYD4*, *RPA1*, *RTN4RL1*, *DPH1*, *OVCA2*, *HIC1*, *SMG6*, *SRR*, *TSR1*, *SGSM2*, *MNT*, *METTL16*, *PAFAH1B1*, *MIR22*, *MIR132*, *MIR212*
10	17q21.31	39,488,528.5	41,074,264.5	*EIF1*, *HAP1*, *JUP*, *LEPREL4*, *FKBP10*, *NT5C3L*, ***ACLY***, *TTC25*, *CNP*, *DNAJC7*, *NKIRAS2***, *ZNF385C***, *HSPB9*, *DHX58*, *KAT2A*, *RAB5C*, *KCNH4*, *HCRT*, *GHDC*, ***STAT5B*****, *STAT5A*, *STAT3***, *PTRF*, *ATP6V0A1*, *NAGLU*, *HSD17B1*, *COASY*, *MLX*, *TUBG1*, *TUBG2*, *PLEKHH3*, *CCR10*, *CNTNAP1*, *EZH1*, *CNTD1*, *BECN1*, *PSME3*, *RAMP2*, *WNK4*, *G6PC*

Note: Regions (genes) of interest are shown in bold.

**Table 5.** Genetic losses with a size of <5 Mbp and high amplitude (log2 ratio T/N < −0.5) .

Tumour No.	Cytoband	Start	End	Gene
4	1p21.3	97,787,876	97,869,625	***DPYD***
7	2p25.1	12,467,758	12,585,765	–
8	2q11.2	98,394,698	98,518,643	*TMEM131*
6	2q37.3	242,514,623	242,656,003	**THAP4*,*** ATG4B*,***DTYMK*,*** ING5
10	4p15.33-4p15.32	13,572,819.5	16,083,385	*CPEB2*, *C1QTNF7*, *CC2D2A*, *FBXL5*, *CD38*, *FAM200B*, *BST1*, *FGFBP1*, *FGFBP2*, *PROM1*
2	4q13.2	69,057,765	69,643,302	*TMPRSS11B*, *YTHDC1*, *TMPRSS11E*, *UGT2B17*, *UGT2B15*
3	5q11.1	49,595,707	50,268,274	*EMB*, *PARP8*
6	5q21.1	97,382,822	97,528,278	–
8	7q36.2	153,161,340	153,255,620	–
6	8p23.2	4,194,881	4,788,752	***CSMD1***
8	8p23.1	7,040,626	7,824,825	*FAM90A5*, *FAM90A7*, *FAM90A8*, *FAM90A9*, *FAM90A10*, *FAM90A13*, *FAM90A14*, *FAM90A18*, *FAM90A19*, *FAM90A20*, ***DEFB4A*****, *DEFB4B*, *DEFB103A*, *DEFB103B*, *DEFB104A*, *DEFB104B*, *DEFB105A*, *DEFB105B*, *DEFB106A*, *DEFB106B*, *DEFB107A*, *DEFB107B*,***SPAG11A*, *SPAG11B*, *ZNF705G*
7	8p23.1	7,226,931	8,117,301	***DEFB4A*, *DEFB4B*, *DEFB103A*, *DEFB103B*, *DEFB104A*, *DEFB104B*, *DEFB105A*, *DEFB105B*, *DEFB106A*, *DEFB106B*, *DEFB107A*, *DEFB107B*,***SPAG11A*, *SPAG11B*, *FAM90A7*, *FAM90A8*, *FAM90A9*, *FAM90A10*, *FAM90A13*, *FAM90A14*, *FAM90A18*, *FAM90A19*, *MIR548I3*
6	8p12-8p11.23	38,276,173	38,625,848	*FGFR1*, *TACC1*
4	10q11.22	47,074,854	47,172,564	*PPYR1*, *ANXA8*, *ANXA8L1*
5	14q21.3	44,332,178	45,975,187	*FSCB*, *KLHL28*, *FAM179B*, *PRPF39*, *FKBP3*, ***FANCM***, *MIS18BP1*
3	14q24.1	68,323,871	68,514,765	***RAD51B***
10	15q11.2	23,013,940.5	23,026,712	*NIPA2*
2	15q13.2	28,606,779	28,865,096	*GOLGA8G*, *GOLGA8F*, *MIR4509-1*, *MIR4509-2*, *MIR4509-3*
6	16p12.1	23,558,024	25,642,549	*UBFD1*, *NDUFAB1*, ***PALB2***, *DCTN5*, *PLK1*, *ERN2*, *CHP2*, *PRKCB*, *CACNG3*, *RBBP6*, *TNRC6A*, *SLC5A11*, *ARHGAP17*, *LCMT1*, *AQP8*, *ZKSCAN2*
7	17q21.31	41,566,570	41,645,009	*DHX8*, *ETV4*
8	18p11.32	1,715,255	1,818,472	–
2	19p13.11-19p12	19,784,330	20,366,322	*ZNF14*, *ZNF506*, *ZNF253*, *ZNF93*, *ZNF682*, *ZNF90*, *ZNF486*
8	19q13.31	48,187,449	48,400,802	***GLTSCR1*, *GLTSCR2*,***EHD2*, *SEPW1*, *TPRX1*, *CRX*, *SULT2A1*
4	Xp11.21	56,489,404	56,532,189	–

Note: Regions (genes) of interest are shown in bold.

Microstructural aberrations were significantly more common in the group of advanced cancers: seven microstructural aberrations per tumour in late-stage tumours, as compared to four microstructural aberrations per tumour in early stage cancers (*p* < 0.006).

Potential candidate oncogenes from regions with copy number changes could include: *CEP72*, *TPPP*, *AHRR*, *EXOC3*, *SLC9A3*, *LOC442126*, *ZDHHC11*, *BRD9*, *TRIP13*, *CLPTM1L*, *SLC6A3* and *LOC401169*. In the analysis of genomic regions with small aberrations, the most common types of aberrations were genetic gains with known role in tumourigenesis: in 7p (containing the oncogene *EGFR*) and 12p (containing the oncogene *KRAS*). We could suggest some new possible candidate oncogenes based on high-amplitude amplification and/or location in the small regions of overlap: *NBPF4* (1p13.3); *ETV1*, *AGR3* and *TSPAN13* (7p21.3-7p21.1); *SOX5* and *FGFR1OP2* (12p12.1-12p11.22); *GPC6* (13q32.1). There were regions of significant genetic losses that may prove useful in identifying potential tumour suppressor genes as possible candidates: *DPYD* (1p21.3); *CLDN22*, *CLDN24*, *ING2*, *CASP3*, *SORBS2* (4q34.2-q35.1); *DEFB* (8p23.1).

Although further studies with a larger sample size would be needed to verify these speculations, our results contribute to the knowledge about the genomic aspects of NSCLC. There has been a great progress in understanding the complex mechanisms of tumourigenesis. Different genetic alterations suggest differences in clinical behaviour and therapeutic response of different tumour subtypes. Owing to the ever increasing opportunities that are open in the genomic era, researchers are able to discover new target therapies specific to each subtype of cancer, and even individual therapy according to the genomic profile of each patient. Molecular profiling of tumours is an important approach in determining prognosis and identifying patients who may respond well to specific therapy. The application of comparative genomic hybridization on DNA microarrays with high resolution allows the establishment of such specificity at chromosome and genetic level, which could help the clinical management of the patients.

## Conclusions

This study is, to the best of our knowledge, the first report on whole genome array CGH analysis in Bulgarian patients diagnosed with primary NSCLC. Comparative genomic hybridization on DNA microarrays with high resolution allows some tumour specifics to be observed at chromosome and genetic level, which could help in the clinical management of the patients. Our results suggested that early stage lung cancers are characterized by large chromosomal aberrations, whereas late-stage tumours harbour microstructural aberrations containing gene amplifications or deletions. Their expression levels are worthy of being investigated as a step towards discovery of new biomarkers. Further studies with a larger sample size would be needed to verify these speculations.
